# Supplementary material for: Mechanistic Insights into the Adenosine A1 Receptor’s Positive Allosteric Modulation for Non-Opioid Analgesics
Source: Cells. 2024 Dec 21;13(24):2121. doi: 10.3390/cells13242121 (PMC11726717; doi:10.3390/cells13242121)
Supplement: Supplementary file 1 [file cells-13-02121-s001.zip › cells-3332605-supplementary.pdf]

Supplementary Information

Mechanistic insights into the adenosine A1 receptor positive allosteric modulation for non-opioid analgesics development.

Tal Weizmann[1], Abigail Pearce[2], Peter Griffin[1], Achille Schild[3], Maren Flaßhoff[3], Philipp Grossenbacher[3], Martin Lochner[3], Christopher A. Reynolds[1], Graham Ladds[2], Giuseppe Deganutti[1]

[1] Centre for Health and Life Sciences, Coventry University, Coventry, CV1 5FB, U.K.

[2] Department of Pharmacology, University of Cambridge, Tennis Court Road, Cambridge, CB2 1PD, U.K.

[3] Institute of Biochemistry and Molecular Medicine, University of Bern, 3012 Bern, Switzerland.

Video S1. Side-by-side comparison between BnOCPA:A_1R:G_{i2} (ternary complex) and BnOCPA:A_1R:G_{i2} (quaternary complex). A_1R is shown as a white transparent ribbon, Gi2 as a transparent red ribbon, BnOCPA as yellow sticks, MIPS521 as orange transparent sticks, and important residues discussed in the manuscript are shown as cyan sticks. For each complex, three MD replicas were merged (3 μs total simulation time).

Table S1. Summary of cAMP assay. Potency (pEC50) and allosteric operator Logαβ are reported for the A_1R agonists CPA, BnOCPA, adenosine, and NECA

		pEC50			Logαβ		
		Mean	SEM	n	Mean	SEM	n
CPA	DMSO	8.5655	0.08839	4	1.3544	0.05865	4
	10μM	10.1843	0.17255	4			
	1μM	9.2085	0.15076	4			
	100nM	8.916	0.09304	4			
	10nM	8.69375	0.09123	4			
BnOCPA	DMSO	7.84775	0.0993	4	1.57347	0.27531	4
	10μM	9.112	0.2495	4			
	1μM	8.79125	0.10214	4			
	100nM	8.053	0.06614	4			
	10nM	7.78525	0.22735	4			

Adenosine	DMSO	7.87	0.05315	4	1.29736	0.2896	4
	10μM	9.43725	0.54466	4			
	1μM	8.489	0.13949	4			
	100nM	8.115	0.1222	4			
	10nM	8.089	0.11782	4			
NECA	DMSO	8.28175	0.14518	4	1.17329	0.18754	4
	10μM	9.6595	0.351	4			
	1μM	8.86225	0.08987	4			
	100nM	8.511	0.13578	4			
	10nM	8.3615	0.16702	4			

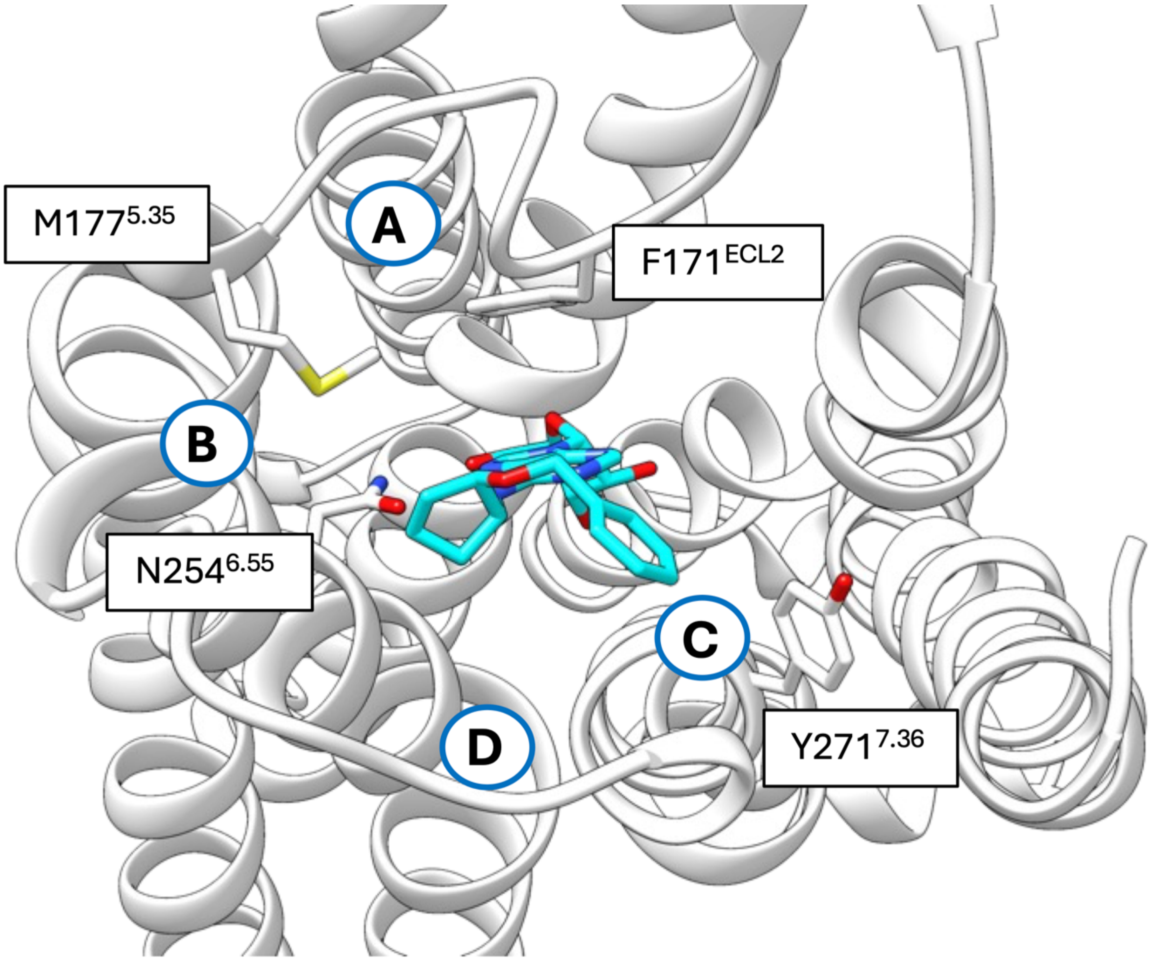

Figure S1. BnOCPA orientations. Four BnOCPA benzyloxy-cyclopentyl adenosine orientations were characterised in our previous work[1]**.** Orientation C (towards TM7) was the starting conformation of the present work (extracellular view, A_1R is represented as a white ribbon).

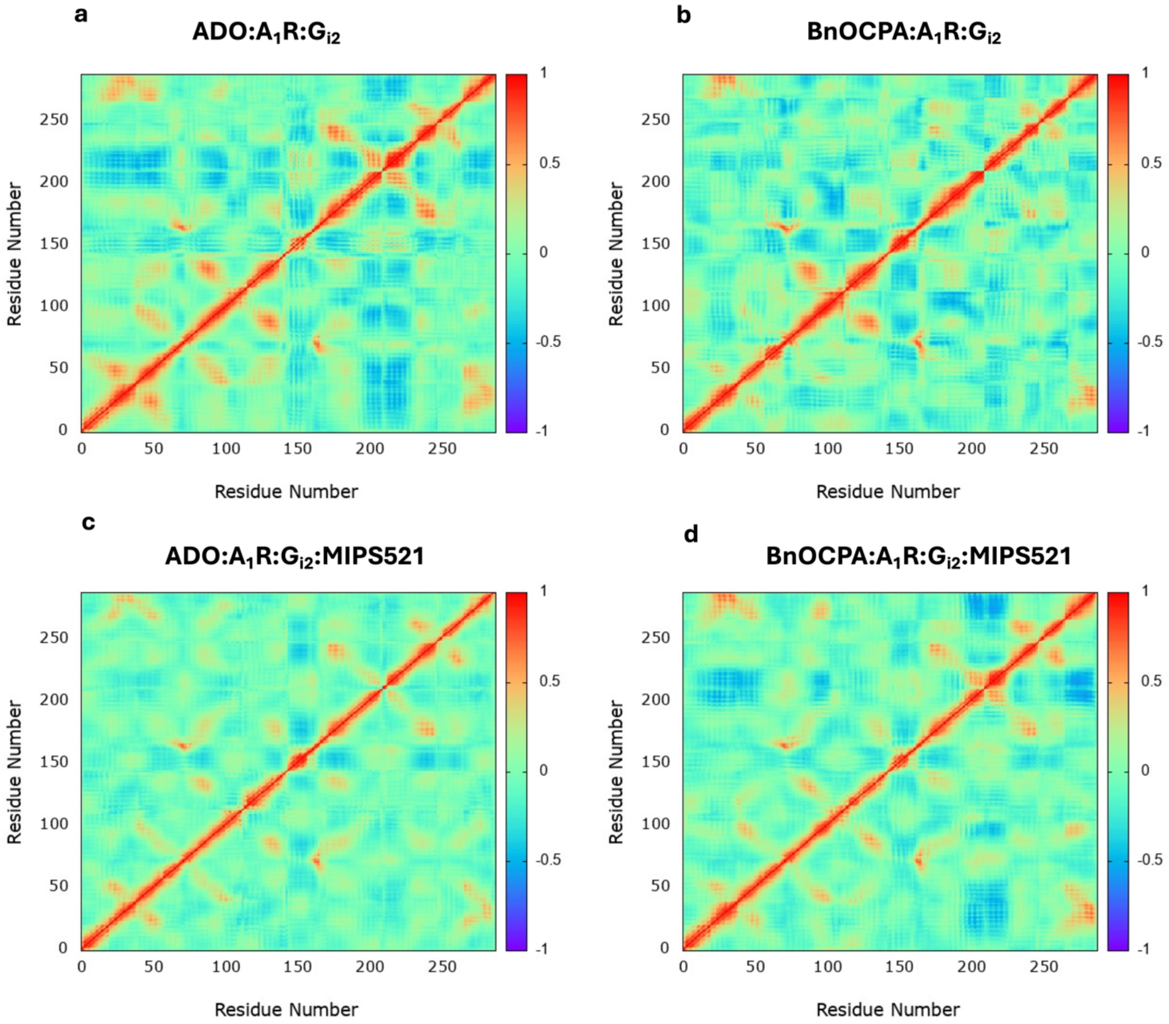

Figure S2. (a-d) Correlation of motion between A_1R residues during MD simulations. Motions correlated are indicated in orange or red, while those anti-correlated are in blue

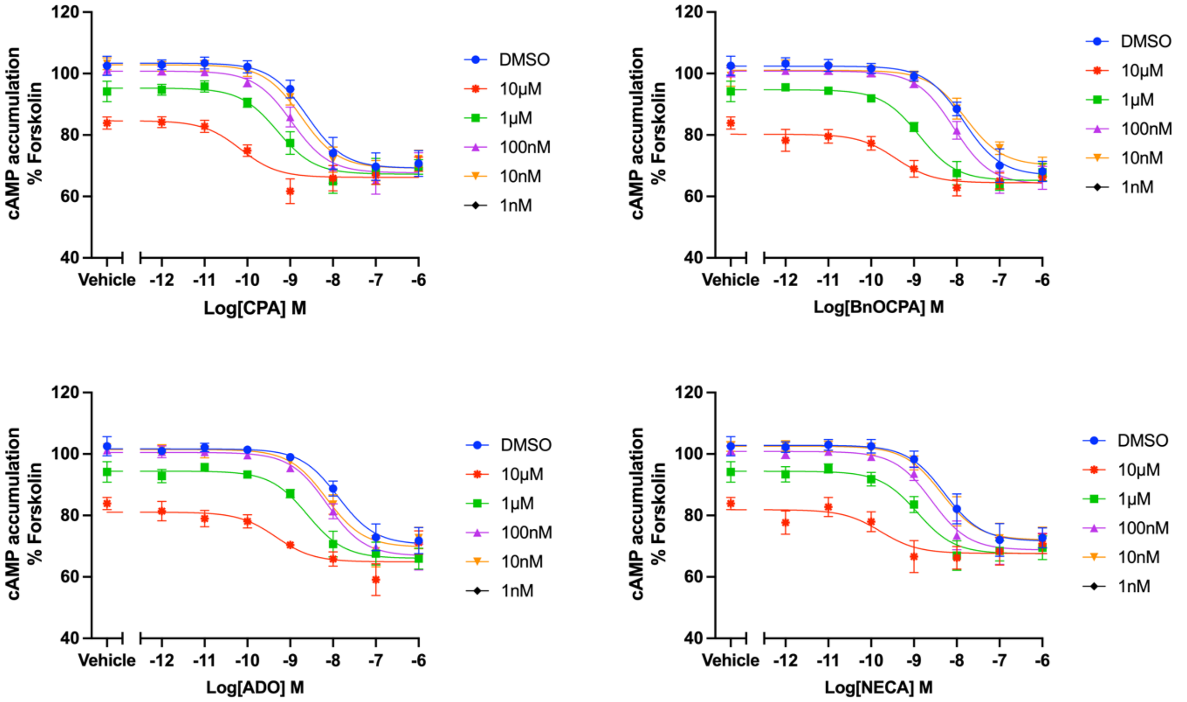

Figure S3. Dose-response curves for cAMP accumulation assay of the A_1R agonists CPA, BnOCPA, adenosine and NECA at different MIPS521 concentrations.

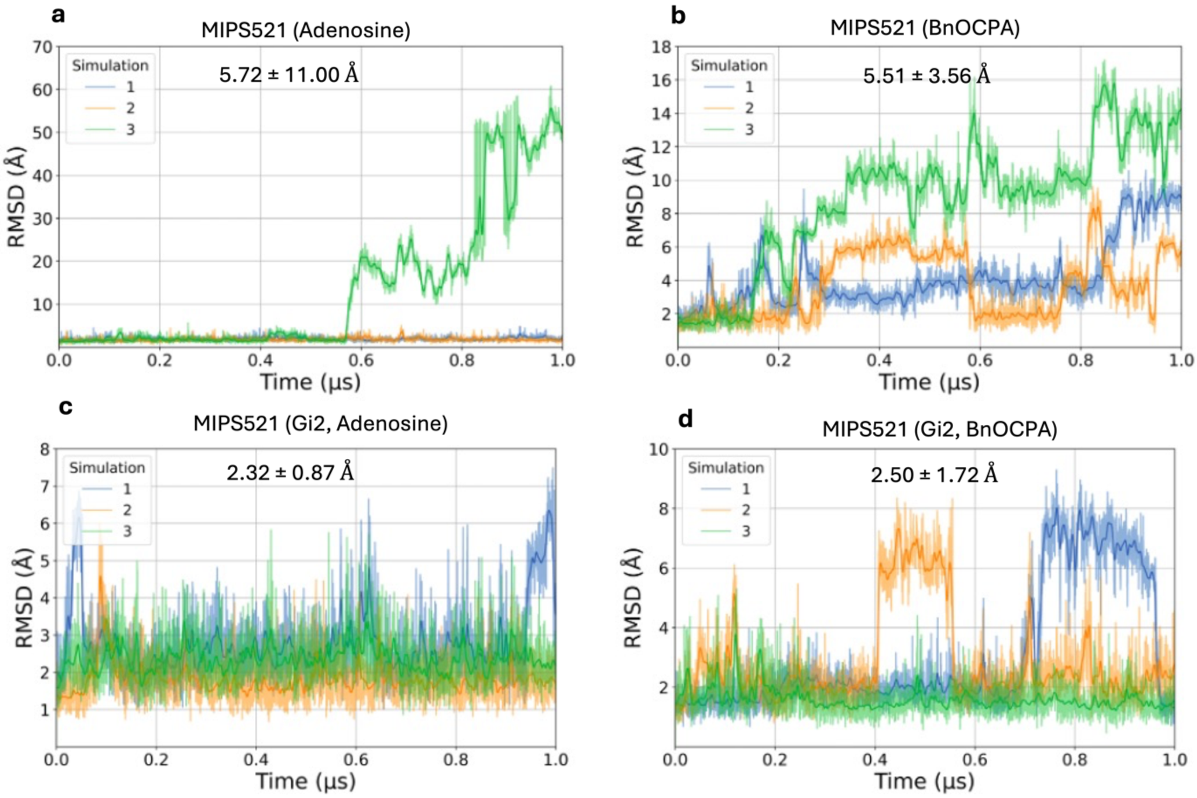

Figure S4. Root mean square deviations (RMSD) of MIPS521 bound to A_1R in complex with adenosine (a), BnOCPA (b), adenosine and G_{i2}, or BnOCPA and G_{i2} (d). MD simulations were performed in triplicate.

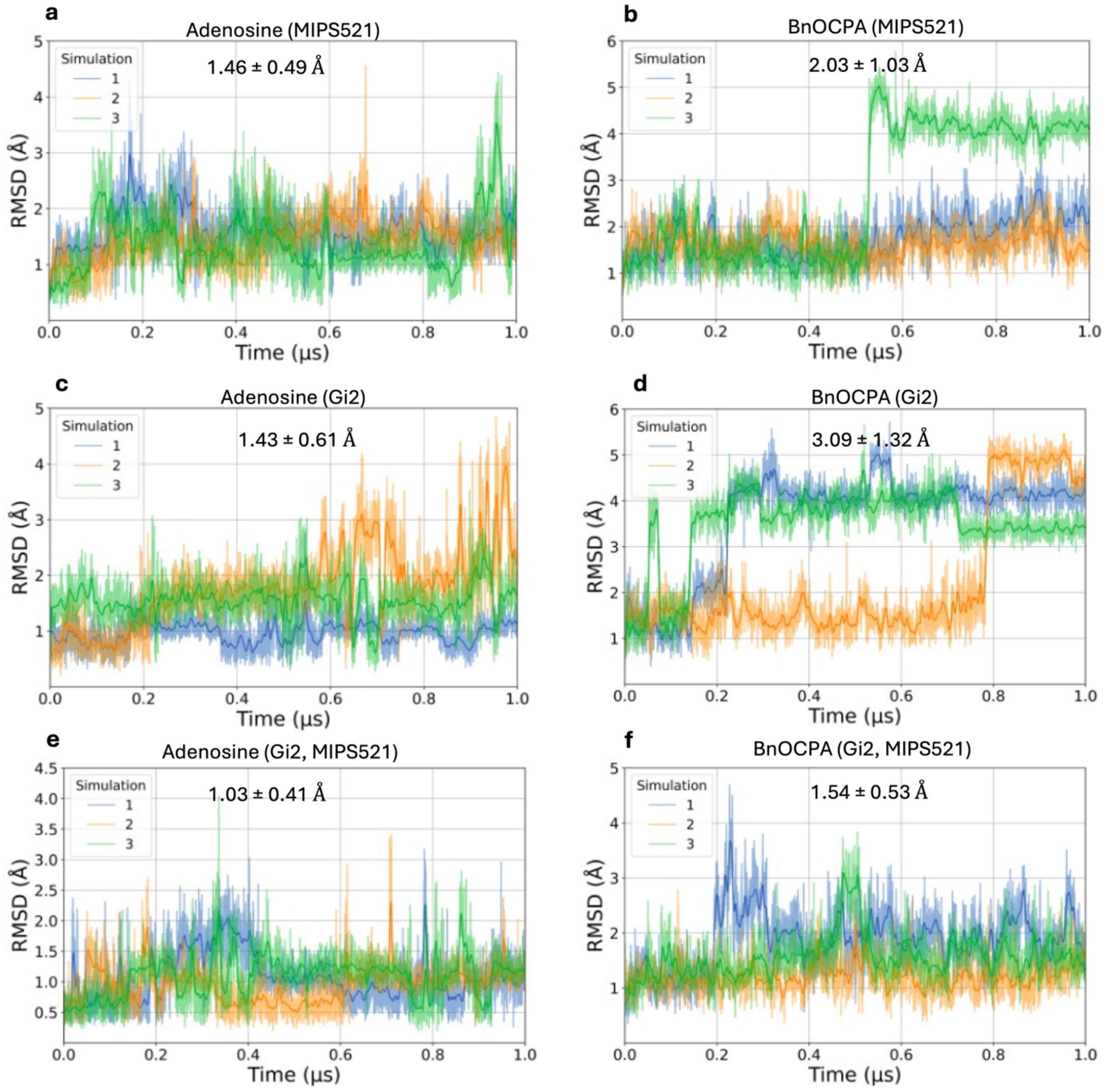

Figure S5. Root mean square deviations (RMSD) of adenosine (a) or BnOCPA (b) bound to A_1R in complex with MIPS521, adenosine (c) or BnOCPA (d) bound to A_1R in complex with G_{i2}, and adenosine (e) or BnOCPA (f) bound to A_1R in complex with G_{i2} and MIPS521. MD simulations were performed in triplicate.

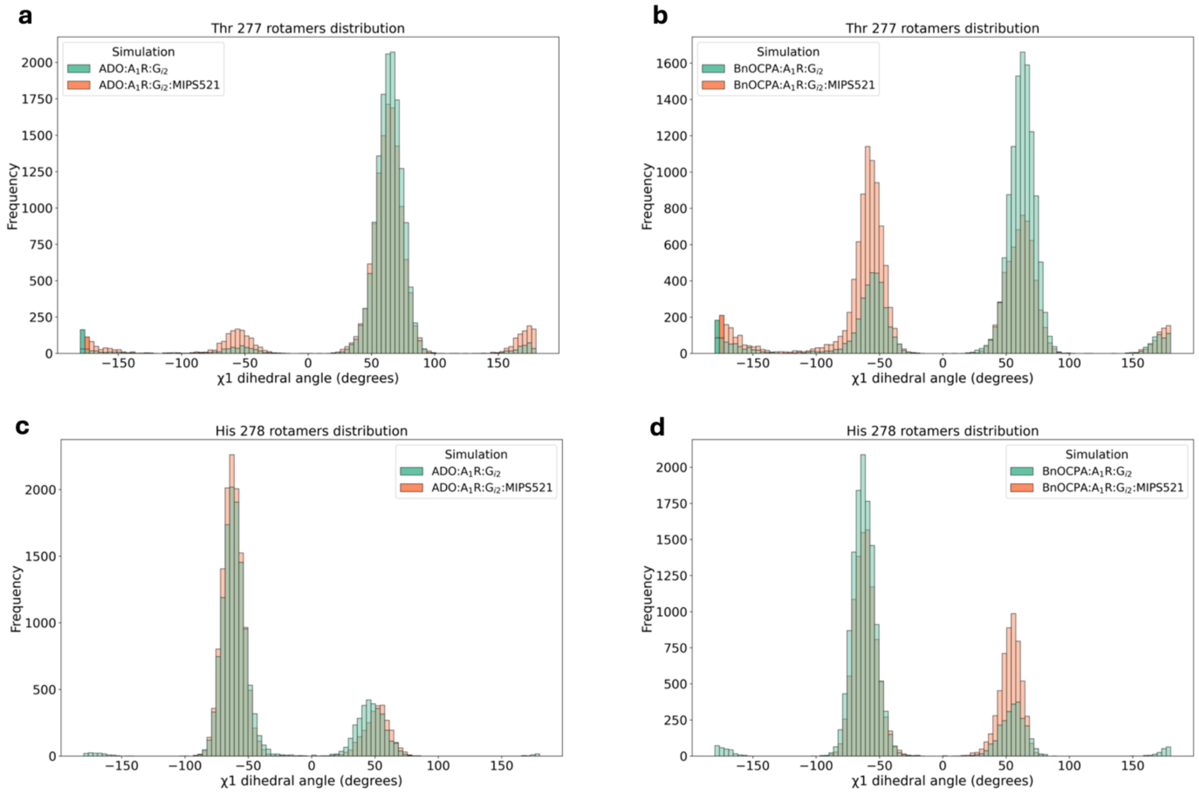

Figure S6. a) and b) Comparison of T277$^{7.42}$ χ1 rotameric distribution during MD simulations of A_1R in complex with a) adenosine, G_{i2} and with or without MIPS521, (b), BnOCPA, G_{i2} and with or without MIPS521. (**c**) and (**d**) Comparison of T278$^{7.43}$ χ1 rotameric distribution during MD simulations of A_1R in complex with c) adenosine, G_{i2} and with or without MIPS521, (d), BnOCPA, G_{i2} and with or without MIPS521.

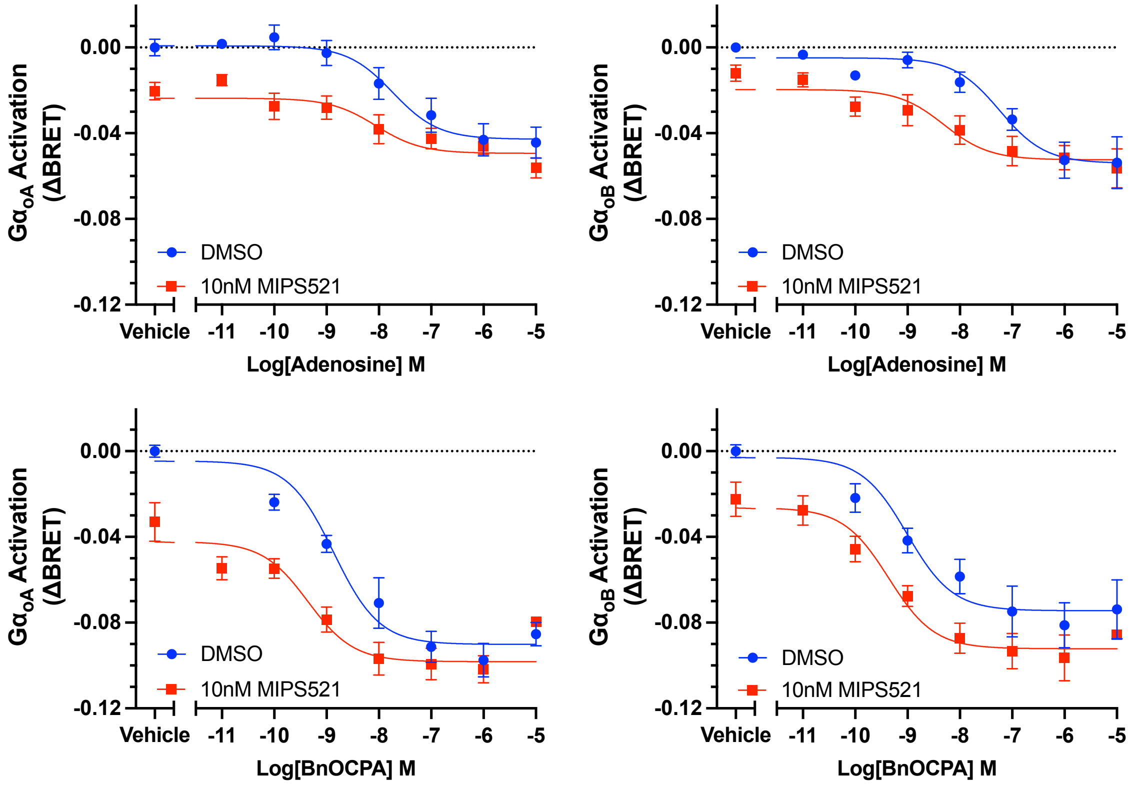

Figure S7. TRUPATH dose-response curves of the A_1R agonists BnOCPA and adenosine in the presence or absence of MIPS521.

Bibliography

1. Wall, M. J. *et al*. Selective activation of Gαob by an adenosine A1 receptor agonist elicits analgesia without cardiorespiratory depression. *Nat. Commun.* **13,** 4150 (2022).
